# Matrix metalloproteinases gene variants and dental caries in Czech children

**DOI:** 10.1186/s12903-020-01130-6

**Published:** 2020-05-12

**Authors:** Petra Borilova Linhartova, Tereza Deissova, Martina Kukletova, Lydie Izakovicova Holla

**Affiliations:** 1 Clinic of Stomatology, Institution Shared with St. Anne’s University Hospital, Faculty of Medicine, Masaryk University, Pekarska 53, 656 91 Brno, Czech Republic; 2 Department of Pathophysiology, Faculty of Medicine, Masaryk University, Brno, Czech Republic

**Keywords:** Polymorphism, Association study, Caries, Oral disease, Genetic predisposition, ELSPAC

## Abstract

**Background:**

Matrix metalloproteinases (MMPs) play an important role in tooth formation and the mineralization of dental tissue. The aim of the study was to analyse Czech children with primary/permanent dentition polymorphisms in those genes encoding MMP2, MMP3, MMP9, MMP13, MMP16, and MMP20, which had been previously associated with dental caries in other populations.

**Methods:**

In total, 782 Czech children were included in this case-control study. DNA samples were taken from 474 subjects with dental caries (with decayed/missing/filled teeth, DMFT ≥ 1) and 155 caries free children (DMFT = 0) aged 13–15 years, as well as 101 preschool children with early childhood caries (ECC, dmft ≥ 1) and 52 caries free children (dmft = 0), were analyzed for nine *MMPs* single nucleotide polymorphisms (SNPs) using real time polymerase chain reaction TaqMan assays.

**Results:**

There were no significant differences in the allele and/or genotype frequencies of all the studied *MMPs *SNPs among children with dental caries in primary/permanent dentition and the healthy controls (*P* > 0.05). In addition, similar allele or genotype frequencies of the studied *MMPs* SNPs were found in children with severe dental caries in their permanent teeth (children with DMFT ≥ 6) and the healthy controls (DMFT = 0, *P* > 0.05).

**Conclusions:**

This study demonstrated the lack of association between the selected SNPs in candidate genes of *MMPs* and the susceptibility to or severity of dental caries in both primary and permanent dentitions in Czech children.

## Background

Dental caries is a multifactorial disease affecting the hard tissues of teeth in both primary and permanent dentitions. In the presence of some carbohydrates, the dental biofilm is dominated by mainly gram-positive carbohydrate-fermenting bacteria causing a pH decrease [1]. In the long-term, frequent exposure to a pH lower than 5.5 causes irreversible demineralization of the tooth enamel with a progression to dentin damage. The demineralization is caused by microbial acids, and the degradation of the dentin organic matrix was thought to have been carried out by microbial proteolytic enzymes. However, the proteases produced by the cariogenic bacteria have been found to be highly pH sensitive, and they are not able to resist the acidic pH fall (pH 4.3) during the demineralization phase [2]. Therefore, the potential role of the host derived proteases, and in particular matrix metalloproteinases (MMPs) in dentine matrix degradation, has been considered [3]. A new concept in caries research termed as “Dentin Degradonomics” has been introduced. This genomic and proteomic approach is applied to the identification of proteases and their substrates in both physiological and pathological conditions [4].

Host MMPs, which are the enzymes responsible for the degradation of intercellular substrates, play an important role in physiological processes during early tooth development [5–7], for example, the *MMP16* gene may regulate ameloblast maturation and enamel formation [8]. Several different types of MMPs have been discovered, among them MMP2 and MMP9 (gelatinase A and B), MMP3 (stromelysin 1), MMP13 (collagenase 3), MMP16 (membrane-type MMP), and MMP20 (enamelysin). MMPs are initially synthesized as inactive zymogens, and can be activated extracellularly by several mechanisms, e.g. low pH [9]. However, host MMPs are functional only in neutral pH. This is maintained by the salivary buffer systems which allow the pH-activated MMPs to degrade the dentin matrix which has been previously demineralized by bacterial acids [10]. The host-derived proteases, and in particular MMPs, may be involved in the destruction of the collagenous dentin matrix and, therefore, in the control or progression of the carious process [3, 11]. MMPs are located throughout the dentine, along the enamel–dentine junction, and in the predentine [12]. Increased MMP presence along the dentine–enamel junction may contribute to the widening of caries along this junction, as it progresses into the dentine [13]. There are various possible sources of MMPs, in carious lesions they are released into the saliva by the salivary glands, into the crevicular fluid by the cells in the gingival crevices and into the dentinal fluids by pulpal odontoblast cells [3]. Inhibition of salivary or dentin endogenous collagenolytic enzymes may provide a preventive means against the progression of caries [4, 14, 15].

Members of MMPs family are encoded by multiple genes and their variability has been widely studied in the context of many diseases including oral diseases (such as oral cancer [16], periodontitis [17], periapical lesions [18], and dental caries [19–30]).

Based on the literature review, the aim of the study was to select the SNPs in the genes encoding MMP2, MMP3, MMP9, MMP13, MMP16, and MMP20, which had been previously associated with dental caries in other populations [19–22, 26–29], and to determine the genotypes of these SNPs in Czech children with primary/permanent dentitions. The null hypothesis is that there is no association between the selected SNPs and dental caries development in both dentitions in our population.

## Methods

### Design of study

This case-control genetic association study was conducted in the period from 2005 to 2018.

All of the procedures performed in the studies involving human participants were in accordance with the ethical standards of the institutional and/or national research committee and with the 1964 Helsinki declaration and its later amendments or comparable ethical standards. Informed consent was obtained from all individual participants included in the study.

### Characteristics of participants and sampling

Two groups of children from the South Moravia region of the Czech Republic were selected according to their dentition - group 1: preschool children with primary dentition, and group 2: children with permanent dentition, all of whom were recruited from the European Longitudinal Study of Pregnancy and Childhood (ELSPAC) [31].

The inclusion criteria for group 1 were: at least 16 primary teeth, general good health, and the willingness of the parents to enter their children in the study. The exclusion criteria for group 1 were: the presence of one or more permanent teeth, a familial relationship between children, and ethnicity other than Caucasian Czech. The inclusion criteria for group 2 were: age 13–15 years, general good health, and the willingness of the parents to enter their children in the study [32]. The exclusion criteria for group 2 were: previous or concomitant therapy with orthodontic appliances, a familial relationship between children, and ethnicity other than Caucasian Czech.

The children included in group 1 were recruited from the out-patients’ department of Paediatric Dentistry, University Hospital Brno, and the Paediatric Department of the Clinic of Stomatology, St. Anne’s Faculty Hospital in Brno, where they were sent for a preventive examination (healthy children) or complex treatment under general anaesthesia (children who were unable to undergo standard dental treatment due to their uncooperativeness and the need for multiple restorations and extractions) in the period 2016–2018. Oral examinations were performed by two mutually calibrated experienced paediatric dentists under standard conditions (using a dental probe and mirror after drying, and in a good light) in a professional dental unit. The dmft index was calculated using dental caries (D_3_ level) as a *cut-off point* for the detection of decay [32, 33].

Children selected from the ELSPAC study (group 2) underwent dental examinations at the Clinic of Stomatology, St. Anne’s University Hospital, in the period 2005–2007. To determine the DMFT index for each child the teeth were first dried and then examined in a good light, all present teeth were examined in the dentist’s chair using a mirror and a dental probe. The data was entered into a standard medical record. A radiograph examination was not performed as it was not part of the routine dental care for these adolescents and would therefore be deemed unethical. The clinical assessment was carried out by one investigator using the cavitation of the lesion as the detection threshold of caries according to the criteria given in the WHO Oral Health Surveys as described previously [34].

To minimize inter-examiner variability in the classification of carious lesions, the participating dentists were trained and calibrated in accurate dmft/DMFT (decayed/missing/filled teeth in primary/permanent dentition) charting. Blood samples from children with early childhood caries (ECC) and samples of buccal epithelial cells from the caries-free children included in group 1, and all of the children from group 2 were collected. The DNA was isolated using the Ultra-Clean^®^BloodSpin^®^ DNA Isolation Kit (Mo Bio Laboratories, Inc., Carlsbad, CA, USA).

### Genetic analysis

The candidate genes encoding MMPs and their specific variants were selected based on associations described previously in other populations, proven functional effects, and/or a minor allele frequency (MAF) higher than 0.1 in the population studied. Determinations of the nine *MMPs* polymorphisms (rs2287074, rs243865, rs679620, rs17576, rs2252070, rs2046315, rs10429371, rs1711437, rs1784418) were based on the polymerase chain reaction using 5′ nuclease TaqMan® assays (C___3225956_10, C___3225943_10, C___3047717_1_, C__11655953_10, C__25474083_10, C__11189892_10, C__30078580_10, C___2158805_20, C___7492661_10, respectively). The reaction mixture and conditions were designed according to the manufacturer’s instructions (Thermo Fisher Scientific, Waltham, MA, USA), and fluorescence was measured using the ABI PRISM 7000 Sequence Detection System. SDS version 1.2.3 software was used to analyze real-time and endpoint fluorescence data.

### Statistical analysis

All statistical analyses were performed using the program package Statistica v. 13.0 (StatSoft, Inc., Tulsa, OK, USA). The Fisher-exact test was used for testing the differences in the allele frequencies, and the chi-square test was used (χ^2^) for calculating the Hardy-Weinberg equilibrium (HWE) and the differences in the genotype frequencies between the cases and controls in each group. Only values of *P* below 0.05 were considered statistically significant. The Bonferroni correction was used to adjust the level according to the number of independent comparisons to the overall value of 0.05.

## Results

In total, 782 generally healthy Czech children were included in this study. Group 1 was comprised of 101 children with ECC (dmft ≥ 1) aged 3.51 ± 0.88 years (mean ± standard deviation), 59 boys and 42 girls and 52 caries-free children with primary dentition (dmft = 0) aged 3.92 ± 1.04 years, there were 24 boys and 28 girls, (*P* > 0.05 both for age and gender distributions). Group 2 consisted of 474 subjects with dental caries (DMFT ≥ 1), 253 boys and 221 girls, and 155 caries-free children with permanent dentition (DMFT = 0), 83 boys and 72 girls, all aged 13–15 years, (*P* > 0.05 both for age and gender distributions). Because the dmft/DMFT indexes were calculated using dental caries (D_3_ level), i.e. cavitated lesions as a cut-off point for the detection of decay, their kappa scores were above 0.95.

The genotype frequencies of all nine studied *MMPs* SNPs were in the HWE in both groups of caries-free children (dmft = 0 and DMFT = 0, *P* > 0.05). There were no significant differences in the allele or genotype frequencies of any of the studied *MMPs* SNPs among the children with dental caries in the primary/permanent dentitions and the healthy controls of the corresponding age group (*P* > 0.05). In addition, similar allele or genotype frequencies of the studied *MMPs* SNPs were found in the children with severe dental caries in permanent teeth (111 children with DMFT ≥ 6) and the healthy controls (DMFT = 0) (*P* > 0.05, see Table 1).

**Table 1:** Genotype frequencies of *MMPs* SNPs in Czech children according to their caries experience

***MMP*** SNP	Genotypes	Caries-free children (dmft = 0)	Children with ECC (dmft ≥ 1)	***P*** value	Caries-free children (DMFT = 0)	Caries-affected children (DMFT ≥ 1)	***P*** value^**#**^	Caries-affected children (DMFT ≥ 6)	***P*** value^**#**^
		***N*** **= 52 (%)**	***N*** **= 101 (%)**		***N*** **= 155 (%)**	***N*** **= 474 (%)**		***N*** **= 111(%)**	
***MMP2*** (rs2287074)	GG	16 (30.8)	31 (30.7)		46 (29.7)	144 (30.4)		31 (27.9)	
	AG	25 (48.0)	56 (55.4)	0.480	77 (49.7)	236 (49.8)	0.971	53 (47.7)	0.774
	AA	11 (21.2)	14 (13.9)		32 (20.6)	94 (19.8)		27 (24.3)	
	A	57 (54.8)	118 (58.4)	0.314	169 (54.5)	524 (55.3)	0.433	115 (51.8)	0.298
	G	47 (45.2)	84 (41.6)		141 (45.5)	424 (44.7)		107 (48.2)	
***MMP2*** (rs243865)	CC	28 (53.8)	62 (61.3)		89 (57.4)	280 (59.0)		68 (61.3)	
	CT	21 (40.4)	34 (33.7)	0.668	58 (37.4)	162 (34.2)	0.647	36 (32.4)	0.681
	TT	3 (5.8)	5 (5.0)		8 (5.2)	32 (6.8)		7 (6.3)	
	C	77 (74.0)	158 (78.2)	0.248	236 (76.1)	722 (76.2)	0.523	172 (77.5)	0.399
	T	27 (26.0)	44 (21.8)		74 (23.9)	226 (23.8)		50 (22.5)	
***MMP3*** (rs679620)	CC	12 (23.1)	24 (23.8)		37 (23.9)	125 (26.4)		31 (27.9)	
	CT	29 (55.8)	55 (54.5)	0.988	77 (49.7)	229 (48.3)	0.824	49 (44.1)	0.642
	TT	11 (21.1)	22 (21.7)		41 (26.4)	120 (25.3)		31 (27.9)	
	C	53 (51.0)	103 (51.0)	0.546	151 (48.7)	479 (50.5)	0.312	111 (50.0)	0.419
	T	51 (49.0)	99 (49.0)		159 (51.3)	469 (49.5)		111 (50.0)	
***MMP9*** (rs17576)	AA	17 (32.7)	37 (36.6)		62 (40.0)	198 (41.8)		51 (45.9)	
	AG	29 (55.8)	54 (53.5)	0.873	74 (47.7)	216 (45.6)	0.894	53 (47.7)	0.237
	GG	6 (11.5)	10 (9.9)		19 (12.3)	60 (12.6)		7 (6.3)	
	A	63 (60.6)	128 (63.4)	0.361	198 (63.9)	612 (64.6)	0.439	155 (69.8)	0.090
	G	41 (39.4)	74 (36.6)		112 (36.1)	336 (35.4)		67 (30.2)	
***MMP13*** (rs2252070)	TT	27 (51.9)	55 (54.5)		78 (50.3)	215 (45.4)		50 (45.1)	
	CT	19 (36.6)	38 (37.6)	0.762	64 (41.3)	208 (43.9)	0.485	47 (42.3)	0.466
	CC	6 (11.5)	8 (7.9)		13 (8.4)	51 (10.7)		14 (12.6)	
	T	73 (70.2)	148 (73.3)	0.330	220 (71.0)	638 (67.3)	0.128	147 (66.2)	0.142
	C	31 (29.8)	54 (26.7)		90 (29.0)	310 (32.7)		75 (33.8)	
***MMP16*** (rs2046315)	GG	34 (65.4)	69 (68.3)		113 (72.9)	353 (74.5)		74 (66.7)	
	AG	15 (28.8)	30 (29.7)	0.458	36 (23.2)	111 (23.4)	0.481	33 (29.7)	0.490
	AA	3 (5.8)	2 (2.0)		6 (3.9)	10 (2.1)		4 (3.6)	
	G	83 (79.8)	168 (83.2)	0.283	262 (84.5)	817 (86.2)	0.261	181 (81.5)	0.214
	A	21 (20.2)	34 (16.8)		48 (15.5)	131 (13.8)		41 (18.5)	
***MMP16*** (rs10429371)	TT	33 (63.5)	51 (50.5)		101 (65.2)	306 (64.5)		63 (56.8)	
	CT	13 (25.0)	43 (42.6)	0.090	44 (28.4)	152 (32.1)	0.203	39 (35.1)	0.380
	CC	6 (11.5)	7 (6.9)		10 (6.4)	16 (3.4)		9 (8.1)	
	T	79 (76.0)	145 (71.8)	0.261	246 (79.4)	764 (80.6)	0.345	165 (74.3)	0.104
	C	25 (24.0)	57 (28.2)		64 (20.6)	184 (19.4)		57 (25.7)	
***MMP20*** (rs1711437)	CC	14 (26.9)	33 (32.7)		47 (30.3)	157 (33.1)		44 (39.7)	
	CT	27 (51.9)	44 (43.6)	0.610	79 (51.0)	224 (47.3)	0.717	53 (47.7)	0.196
	TT	11 (21.2)	24 (23.7)		29 (18.7)	93 (19.6)		14 (12.6)	
	C	55 (52.9)	110 (54.5)	0.444	173 (55.8)	538 (56.8)	0.410	141 (63.5)	0.045*
	T	49 (47.1)	92 (45.5)		137 (44.2)	410 (43.2)		81 (36.5)	
***MMP20*** (rs1784418)	CC	16 (30.7)	32 (31.7)		46 (29.7)	148 (31.2)		42 (37.8)	
	CT	24 (46.2)	46 (45.5)	0.993	84 (54.2)	234 (49.4)	0.521	54 (48.7)	0.372
	TT	12 (23.1)	23 (22.8)		25 (16.1)	92 (19.4)		15 (13.5)	
	C	56 (53.8)	110 (54.5)	0.507	176 (56.8)	530 (55.9)	0.421	138 (62.2)	0.124
	T	48 (46.2)	92 (45.5)		134 (43.2)	418 (44.1)		84 (37.8)	

#compared with caries-free children (DMFT = 0)

*P_corr_ > 0.05

The MAF of all of the nine studied *MMPs* SNPs were similar in the European population (EUR, *N* = 1006, data obtained from NCBI database) and the Czech population (*P* > 0.05, see Table 2). The highest difference in MAFs was present in *MMP16* SNP (rs2046315) (6.4% between EUR population and Czech children with dmft = 0). In contrast to the EUR population, the MAFs of specific *MMPs* SNPs in Bulgarians, Turks, Brazilians, Chinese, and Saudi Arabians are more or less frequent (difference to 22.7%). Also, the allele distribution of *MMP20* (rs1784418) in Polish Caucasians differs in frequency from the EUR population (difference to 7.6%), while similar frequencies were found in both *MMP16* SNPs (rs2046315 and rs10429371) in COHRA1, including Caucasian northern Appalachian families and the EUR population, see Table 2.

**Table 2:** *MMPs* SNPs in caries-free controls from this study and other caries-free controls in different populations

***MMP*** SNP	***MMP*** SNP localization	Minor allele	MAF				References
			***N*** = 1006 EUR (NCBI)^b^	***N*** = 52 Czech (dmft = 0)	***N*** = 155 Czech (DMFT = 0)	Caries-free subjects (N nationality, index^a^)	
**MMP2** (rs2287074)	exon 9	A	43.3%	45.2%	45.5%	52.5% (20 Bulgarians, DMFT)	[19]
**MMP2** (rs243865)	promotor	T	25.9%	26.0%	23.9%	24.3% (212 Brazilians, dmft/DMFT)	[20]
						19.0% (100 Saudi Arabians, DMFT)	[21]
***MMP3*** (rs679620)	exon 2	T	47.2%	49.0%	51.3%	57.5% (20 Bulgarians, DMFT)	[19]
***MMP9*** (rs17576)	exon 6	G	38.1%	39.4%	36.1%	31.0% (211 Brazilians, dmft/DMFT)	[20]
						37.5% (100 Saudi Arabians, DMFT)	[20]
						29.2% (274 Brazilians, dmft)	[22]
***MMP13*** (rs2252070)	promotor	C	29.9%	29.8%	29.0%	36.1% (208 Brazilians, dmft/DMFT)	[20]
						38.7% (97 Saudi Arabians, DMFT)	[21]
						31.9% (216 Brazilians, dmft/DMFT)	[23]
						52.6% (196 Chinese, DMFT)	[24]
***MMP16*** (rs2046315)	intergenic	A	13.8%	20.2%	15.5%	13% (1769 COHRA1, dft/DMFT)^b^	[25]
***MMP16*** (rs10429371)	intergenic	C	21.7%	24.0%	20.6%	22% (1769 COHRA1, dft/DMFT)^b^	[25]
***MMP20*** (rs1711437)	intron	T	42.6%	47.1%	44.2%	38.3% (450 Brazilians, dmft)	[22]
***MMP20*** (rs1784418)	intron 1	T	45.1%	46.2%	43.2%	39.8% (457 Brazilians, dmft)	[22]
						51.2% (216 Brazilians, dmft/DMFT)	[23]
						43.8% (161 Brazilians, dmft/DMFT)	[26]
						40.7% (259 Turks, dmft)^b^	[27]
						52.9% (68 Brazilians, dmft)	[28]
						37.5% (48 Poles, dt)	[29]

The localization, function and MAF in European population according to NCBI for selected *MMPs* SNPs

^a^index for evaluation of caries status (dt, dft or dmft for primary dentition, DMFT for permanent dentition), ^b^whole population, not only caries-free controls

## Discussion

The progression of carious lesions differs between the primary and permanent dentitions as a result of the different anatomical and morphological characteristics. Wang et al. [35] suggested that the susceptibility to the development of dental caries in the primary dentition has a different genetic background than in permanent dentition.

Host-derived MMPs can originate both from saliva and dentin, and are activated in an acid environment, thus they are able to digest the demineralized dentin matrix. Human MMP2, MMP9, and MMP8 (collagenase 2) were identified in demineralized dentinal lesions [9]; MMP3 was also present in human dentine [36]. The odontoblasts, the main cellular source of MMP20 in the dentin-pulp complex, secrete this enzyme into the dentinal fluid [37]. The MMPs levels can be influenced by many factors, such as growth factors, cytokines, and hormones (oestrogen), as well as gene variability leading to changes in their gene expression and/or protein functions. Therefore, this study focused on variants in the *MMPs* genes and their possible role in the pathogenesis of dental caries in primary and permanent dentitions.

Raivisto et al. [30] studied several SNPs in the genes encoding MMP2, MMP3, MMP8, MMP9, and MMP13, and reported no association with dental caries or initial carious lesions in 94 Finnish adults. The results of our multigene SNPs analysis in a larger population sample (625 Czech children with permanent teeth) are in line with their findings. In Czech preschool children with primary dentition, the selected *MMPs* SNPs were not associated with dental caries either.

Price et al. [38] suggested that common *MMP2* SNP (rs243865) located in the CCACC box of the Sp1-binding site influences its gene transcription, thus this variant is a candidate for further studies of pathological processes. Although the frequencies of the AA genotype *MMP2* SNP (rs2287074) were previously found significantly higher in Bulgarian students with low caries experience (DMFT from 1 to 4) than in caries-free subjects (DMFT = 0), the size of the studied sample was small (41 vs. 20 subjects, respectively) [19]. Apart from the findings in the study by Karayasheva et al. [19], no other *MMP2* gene variants (rs243847 and rs243865) were associated with dental caries [20–22], which is in accordance with our results.

Controversial results have been recorded in *MMP3* SNP (rs679620) and its relation to dental caries. A Brazilian study which dealt with this variant in a large cohort of children with primary dentition did not find any association with dental caries [22], similar to the afore-mentioned Finnish study [30]. While Bulgarian students with the CC genotype suffered significantly more frequently from dental caries (DMFT ≥ 1), CC genotype frequencies between caries-free adults and the group of patients with high caries experience in permanent dentition (DMFT ≥ 5) were the same [19]. However, the authors stated that the presence of the CC genotype decreased the risk of developing a high number of caries by about four times [19]. We determined similar allele and genotype frequencies between the studied groups of Czech children, even in comparison with extreme phenotypes (caries-free subjects vs. children with high caries experience DMFT ≥ 6).

The GG genotype of *MMP9* SNP (rs17576) has previously been associated with a lower risk of dental caries in the primary dentition [22]. In the multivariate analysis, the AA genotype was found to be a risk factor for dental caries when the variable defining dental status was comprised from the dmft index and white spot lesion (WSL) together, but not separately [22]. *MMP9* and *MMP13* SNPs (rs17576 and rs2252070, respectively) were monitored in studies by Tannure et al. [20] and Alyousef et al. [21]. The same conclusion about the negative association with dental caries was reached in cases of *MMP9* variants, in agreement with our results. However, in the case of *MMP13* SNP there is a discrepancy in the hypothesised functional importance [39]. The CC genotype decreased the risk of dental caries in Brazilians aged 3–21 years [20]. However, another Brazilian study involving 10–12 year old children found no association between this *MMP13* SNP (rs2252070) and dental caries [23]. Moreover, the distributions of the alleles and genotypes of *MMP13* SNP (rs2252070) were similar between the cases and controls in children aged 5–13 years from Saudi Arabia [21], and in both groups of Czech children. Additionally, no significant differences were found recently in the alleles or genotypes of *MMP13* between 12 and 15 year old children with and without dental caries in China [24].

In total, 889 children and 2698 adults from different cohorts were involved in the genome-wide association study by Lewis et al. [25] Almost 30 genetic variants spanning the *MMP10*, *MMP14*, and *MMP16* genes were investigated. Only *MMP16* SNPs (rs2046315 and rs10429371) were significantly associated with dental caries in an individual sample of Caucasian adults, and via meta-analysis across 8 adult samples after gene-wise adjustment for multiple comparisons [25]. In contrast, no differences were observed in the allele or genotype frequencies according to caries status between Czech children with primary and permanent dentitions.

Antunes et al. [22] associated *MMP20* SNP (rs1711437) with the presence of WSL, but not with the dmft index, in a cohort of 786 children aged from 2 to 6 years in Brazil. Our results are largely in agreement with these findings, however, we did not evaluate the WSL. The meta-analysis by Filho et al. [28] showed the association of *MMP20* SNP (rs1784418) with dental caries in the primary dentition only for Brazilians and not for the Turkish population. However, Vasconcelos et al. [23] found no association between this SNP and dental caries of mixed dentition in Brazilian children. In line with the study by Gerreth et al. [29], which included 96 Polish children aged 20–42 months, *MMP20* SNP (rs1784418) was not associated with dental caries in the primary dentition in Czech children.

There are some limitations to our study, such as the risk of false-negative results in a case-control designed study with a relatively small number of caries-free preschool children, as well as no determination of *MMPs* mRNA or protein levels. On the other hand, this is the first study to analyse several *MMPs* polymorphisms in relation to both dmft and DMFT scores in a European Caucasian population. Minor allele frequencies of all studied *MMPs* SNPs in generally healthy and caries-free Czech children are in concordance with data referred to in the NCBI for the European population (EUR) (see Table 2). The EUR population mentioned in Table 2 is from the 1000 Genomes Project super population [40] and includes Utah Residents (CEPH) with Northern and Western European Ancestry (CEU), the Toscani in Italia (TSI), the Finnish in Finland (FIN), the British in England and Scotland (GBR) and the Iberian Population in Spain (IBS). An explanation of the discrepancies between our findings and other non-Caucasian studies may probably be due to the interpopulation heterogeneity. The allele and genotype frequencies were compared within each group between the cases and controls, therefore, there is no bias in the statistical evaluation according to the different periods of the subject recruitment.

Although *MMPs* are considered as candidate genes for dental caries, we assume that individual variants have a small effect on it and a complex research approach to this multifactorial disease is preferable. Further research in larger populations with different ethnic backgrounds should be carried out before the exclusion of these *MMPs* SNPs as risk factors in the pathogenesis of dental caries.

## Conclusions

In conclusion, the genotypes in selected *MMPs* SNPs in Czech children were determined and this study demonstrated the lack of association between the selected *MMPs* gene variants and the susceptibility to or severity of dental caries in both primary and permanent dentitions in the Czech population.

## Abbreviations

dmft/DMFT: Decayed/missing/filled teeth in primary/permanent dentition
ECC: Early childhood caries
ELSPAC: European Longitudinal Study of Pregnancy and Childhood
EUR: European population
MAF: Minor allele frequency
MMP: Matrix metalloproteinase
SNP: Single nucleotide polymorphism
WSL: White spot lesion

## References

[1] Larsen T, Fiehn NE (2017). Dental biofilm infections - an update. APMIS..

[2] Kawashaki K, Featherstone JD (1997). Effects of collagenase on root demineralization. J Dent Res.

[3] Jain A, Bahuguna R (2015). Role of matrix metalloproteinases in dental caries, pulp and periapical inflammation: an overview. J Oral Biol Craniofac Res.

[4] Tjäderhane L, Buzalaf MA, Carrilho M, Chaussain C (2015). Matrix metalloproteinases and other matrix proteinases in relation to cariology: the era of 'dentin degradomics'. Caries Res.

[5] Lu Y, Papagerakis P, Yamakoshi Y, Hu JC, Bartlett JD, Simmer JP (2008). Functions of KLK4 and MMP-20 in dental enamel formation. Biol Chem.

[6] Sahlberg C, Reponen P, Tryggvason K, Thesleff I (1999). Timp-1, −2 and −3 show coexpression with gelatinases a and B during mouse tooth morphogenesis. Eur J Oral Sci.

[7] Feng J, McDaniel JS, Chuang HH, Huang O, Rakian A, Xu X, Steffensen B, Donly KJ, MacDougall M, Chen S (2012). Binding of amelogenin to MMP-9 and their co-expression in developing mouse teeth. J Mol Histol.

[8] Liu C, Niu Y, Zhou X, Xu X, Yang Y, Zhang Y, Zheng L (2015). Cell cycle control, DNA damage repair, and apoptosis-related pathways control pre-ameloblasts differentiation during tooth development. BMC Genomics.

[9] Tjäderhane L, Larjava H, Sorsa T, Uitto VJ, Larmas M, Salo T (1998). The activation and function of host matrix metalloproteinases in dentin matrix breakdown in caries lesions. J Dent Res.

[10] Chaussain C, Boukpessi T, Khaddam M, George A, Menashi S (2013). Dentin matrix degradation by host matrix metalloproteinases: inhibition and clinical perspectives toward regeneration. Front Physiol.

[11] Chaussain-Miller C, Fioretti F, Goldberg M, Menashi S (2006). The role of matrix metalloproteinases (MMPs) in human caries. J Dent Res.

[12] Boushell LW, Kaku M, Mochida Y, Bagnell R, Yamauchi M (2008). Immunohistochemical localization of matrix metalloproteinase-2 in human coronal dentin. Arch Oral Biol.

[13] Moon PC, Weaver J, Brooks CN (2010). Review of matrix metalloproteinases. Effect on the hybrid dentin bond layer stability and chlorhexidine clinical use. Open Dent J.

[14] Femiano F, Femiano R, Femiano L, Jamilian A, Rullo R, Perillo L (2016). Dentin caries progression and the role of metalloproteinases: an update. Eur J Paediatr Dent.

[15] Mazzoni A, Tjäderhane L, Checchi V, Di Lenarda R, Salo T, Tay FR, Pashley DH, Breschi L (2015). Role of dentin MMPs in caries progression and bond stability. J Dent Res.

[16] Pereira AC, Dias do Carmo E, Dias da Silva MA (2012). Blumer Rosa LE. Matrix metalloproteinase gene polymorphisms and oral cancer. J Clin Exp Dent.

[17] Li W, Zhu Y, Singh P, Ajmera DH, Song J, Ji P (2016). Association of common variants in MMPs with periodontitis risk. Dis Markers.

[18] Menezes-Silva R, Khaliq S, Deeley K, Letra A, Vieira AR (2012). Genetic susceptibility to periapical disease: conditional contribution of MMP2 and MMP3 genes to the development of periapical lesions and healing response. J Endod.

[19] Karayasheva D, Glushkova M, Boteva E, Mitev V, Kadiyska T (2016). Association study for the role of matrix metalloproteinases 2 and 3 gene polymorphisms in dental caries susceptibility. Arch Oral Biol.

[20] Tannure PN, Küchler EC, Falagan-Lotsch P, Amorim LM, Raggio Luiz R, Costa MC, Vieira AR, Granjeiro JM (2012). MMP13 polymorphism decreases risk for dental caries. Caries Res.

[21] Alyousef YM, Borgio JF, AbdulAzeez S, Al-Masoud N, Al-Ali AA, Al-Shwaimi E, Al-Ali AK (2017). Association of MBL2 gene polymorphism with dental caries in Saudi children. Caries Res.

[22] Antunes LA, Antunes LS, Küchler EC, Lopes LB, Moura A, Bigonha RS, Abreu FV, Granjeiro JM, de Amorim LM, Paixão IC (2016). Analysis of the association between polymorphisms in MMP2, MMP3, MMP9, MMP20, TIMP1, and TIMP2 genes with white spot lesions and early childhood caries. Int J Paediatr Dent.

[23] Vasconcelos KR, Arid J, Evangelista S, Oliveira S, Dutra AL, Silva LAB, Segato RAB, Vieira AR, Nelson-Filho P, Küchler EC (2019). MMP13 contributes to dental caries associated with developmental defects of enamel. Caries Res.

[24] Hu XP, Song TZ, Zhu YY, Wu LL, Zhang X, Zhou JY, Li ZQ (2019). Association of ENAM, TUFT1, MMP13, IL1B, IL10 and IL1RN gene polymorphism and dental caries susceptibility in Chinese children. J Int Med Res.

[25] Lewis DD, Shaffer JR, Feingold E, Cooper M, Vanyukov MM, Maher BS, Slayton RL, Willing MC, Reis SE, McNeil DW, Crout RJ, Weyant RJ, Levy SM, Vieira AR, Marazita ML (2017). Genetic association of MMP10, MMP14, and MMP16 with dental caries. Int J Dent.

[26] Tannure PN, Küchler EC, Lips A, Costa Mde C, Luiz RR, Granjeiro JM, Vieira AR (2012). Genetic variation in MMP20 contributes to higher caries experience. J Dent.

[27] Abbasoğlu Z, Tanboğa İ, Küchler EC, Deeley K, Weber M, Kaspar C, Korachi M, Vieira AR (2015). Early childhood caries is associated with genetic variants in enamel formation and immune response genes. Caries Res.

[28] Filho AV, Calixto MS, Deeley K, Santos N, Rosenblatt A, Vieira AR (2017). MMP20 rs1784418 protects certain populations against caries. Caries Res.

[29] Gerreth K, Zaorska K, Zabel M, Borysewicz-Lewicka M, Nowicki M (2017). Chosen single nucleotide polymorphisms (SNPs) of enamel formation genes and dental caries in a population of polish children. Adv Clin Exp Med.

[30] Raivisto T, Heikkinen A, Kovanen L, Ruokonen H, Kettunen K, Tervahartiala T, Haukka J, Sorsa T (2018). SNP analysis of caries and initial caries in Finnish adolescents. Int J Dent.

[31] Piler P, Kandrnal V, Kukla L, Andrýsková L, Švancara J, Jarkovský J, Dušek L, Pikhart H, Bobák M, Klánová J (2017). Cohort Profile: The European Longitudinal Study of Pregnancy and Childhood (ELSPAC) in the Czech Republic. Int J Epidemiol.

[32] Borilova Linhartova P, Kastovsky J, Bartosova M, Musilova K, Zackova L, Kukletova M, Kukla L, Izakovicova HL (2016). ACE insertion/deletion polymorphism associated with caries in permanent but not primary dentition in Czech children. Caries Res.

[33] Borilova Linhartova P, Deissova T, Musilova K, Zackova L, Kukletova M, Kukla L, Izakovicova HL (2018). Lack of association between ENAM gene polymorphism and dental caries in primary and permanent teeth in Czech children. Clin Oral Investig.

[34] Volckova M, Borilova Linhartova P, Trefna T, Vlazny J, Musilova K, Kukletova M, Kukla L, Izakovicova HL (2014). Lack of association between lactotransferrin polymorphism and dental caries. Caries Res.

[35] Wang X, Shaffer JR, Weyant RJ, Cuenco KT, DeSensi RS, Crout R, McNeil DW, Marazita ML (2010). Genes and their effects on dental caries may differ between primary and permanent dentitions. Caries Res.

[36] Mazzoni A, Papa V, Nato F, Carrilho M, Tjäderhane L, Ruggeri A, Gobbi P, Mazzotti G, Tay FR, Pashley DH, Breschi L (2011). Immunohistochemical and biochemical assay of MMP-3 in human dentine. J Dent.

[37] Sulkala M, Larmas M, Sorsa T, Salo T, Tjäderhane L (2002). The localization of matrix metalloproteinase-20 (MMP-20, enamelysin) in mature human teeth. J Dent Res.

[38] Price SJ, Greaves DR, Watkins H (2001). Identification of novel, functional genetic variants in the human matrix metalloproteinase-2 gene: role of Sp1 in allele-specific transcriptional regulation. J Biol Chem.

[39] Yoon S, Kuivaniemi H, Gatalica Z, Olson JM, Butticè G, Ye S, Norris BA, Malcom GT, Strong JP, Tromp G (2002). MMP13 promoter polymorphism is associated with atherosclerosis in the abdominal aorta of young black males. Matrix Biol.

[40] The International Genome Sample Resource. Available from: http://www.1000genomes.org/category/frequently-asked-questions/population. 2020. Accessed March 15^th^, 2020.

